# When Myocarditis Masquerades as ST-Elevation Myocardial Infarction: A Case of Coxsackie B-induced Acute Heart Failure With Rapid Recovery

**DOI:** 10.7759/cureus.99451

**Published:** 2025-12-17

**Authors:** Afnan Chaudhry, Andrew Mangente, Celebi Yildirim, Mohammad Alazzeh, Gary Ledley

**Affiliations:** 1 Internal Medicine, Tower Health Medical Group, Phoenixville, USA; 2 Cardiology, Tower Health Medical Group, Phoenixville, USA

**Keywords:** acute heart failure (ahf), coxsackie virus, myocarditis, rapid recovery, st-elevation myocardial infarction (stemi)

## Abstract

Coxsackie B viral myocarditis is a rare but important mimic of ST-elevation myocardial infarction (STEMI) and can present with fulminant heart failure and shock. Swift recognition is vital, as supportive therapy alone can lead to rapid recovery. A 69-year-old man with a history of only hypertension presented with dyspnea and fever, despite outpatient antibiotics for pneumonia. On arrival, he was hypotensive and in severe respiratory distress, requiring intubation and norepinephrine. EKG revealed anterolateral ST-segment elevations, and echocardiography showed profoundly depressed left ventricular function (ejection fraction: 10-15%) with regional wall motion abnormalities. Troponin rose to 1,700 ng/L and leukocytes to 17,900/µL. Emergent angiography demonstrated non-obstructive coronaries, raising concern for myocarditis, sepsis-induced cardiomyopathy, or Takotsubo syndrome. CT pulmonary angiography showed bilateral lung infiltrates. Given instability, cardiac MRI and biopsy were deferred. Viral serology was pursued and returned positive for Coxsackie B1-B6. Treatment continued with empiric antibiotics, vasopressors, and intra-aortic balloon pump support. By hospital day four, there was striking clinical recovery, with repeat echocardiography confirming normalization of ventricular function, eliminating the indication for defibrillator therapy. STEMI presentations with non-obstructive coronaries should prompt consideration of alternative etiologies. When cardiac MRI or biopsy is not feasible, Coxsackie B myocarditis can be identified serologically. In these cases, transient circulatory support may suffice given the potential for rapid recovery. Early follow-up echocardiography is crucial to confirm recovery and prevent unnecessary wearable or implantable defibrillator placement. Coxsackie B myocarditis can mimic STEMI with cardiogenic shock. Serology provides a practical diagnostic pathway when MRI and biopsy are not possible. Supportive management may enable rapid reversal of ventricular dysfunction, underscoring the role of early follow-up echocardiography in guiding device therapy decisions.

## Introduction

Myocarditis is an inflammatory disorder of the myocardium most often triggered by viral infections. Common causative pathogens include enteroviruses such as Coxsackievirus B, adenovirus, parvovirus B19, human herpesvirus 6, Epstein-Barr virus, SARS-CoV-2, influenza, and HIV, with Coxsackievirus B being a well-recognized cause of both subclinical and fulminant myocarditis [1-3]. A major clinical challenge arises when myocarditis presents with chest pain, elevated troponin, and regional ST-segment elevation. These findings closely resemble ST-elevation myocardial infarction (STEMI), frequently prompting urgent coronary angiography and complicating early diagnostic clarity [4].

Coxsackie B virus and COVID-19-associated myocarditis are particularly associated with rapid hemodynamic deterioration and severe systolic dysfunction that can closely mimic acute ischemic myocardial injury [2]. Although recovery typically occurs over weeks to months, rapid restoration of ventricular function can occur, adding further complexity to decisions regarding temporary defibrillator support and the need for implantable cardioverter-defibrillator (ICD) placement [3,5,6]. Systemic symptoms such as fever or recent viral illness may raise suspicion for myocarditis, but remain non-specific. While cardiac MRI and biopsy can assist in diagnosis, they are not always feasible in unstable patients. In such situations, serologic testing becomes a valuable tool, particularly when coronary angiography fails to reveal obstructive disease [7].

We report a case of Coxsackie B myocarditis initially presenting as STEMI and complicated by acute heart failure and cardiogenic shock. Supportive therapy resulted in rapid and complete recovery of left ventricular function within days. This case underscores the importance of a broad differential diagnosis in acute cardiac presentations and highlights the potential for full recovery with timely and appropriate management.

## Case presentation

**Figure 1:**
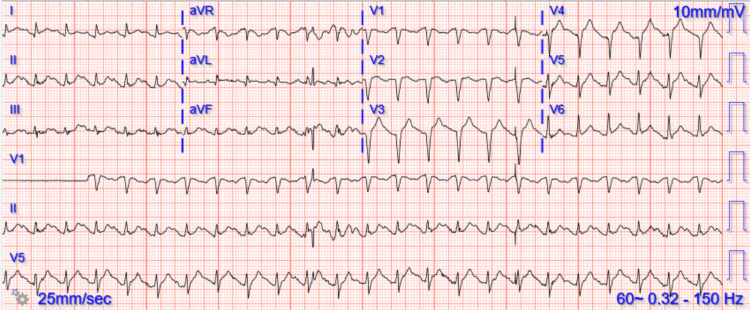
Presenting EKG. Marked ST-segment elevation is visible in the anterolateral leads, consistent with acute transmural ischemia involving the left anterior descending and diagonal coronary territories.

A 69-year-old man with a history of hypertension and glaucoma presented with progressive dyspnea, fatigue, and fevers. He had recently received a course of outpatient antibiotics for presumed community-acquired pneumonia, without improvement. On arrival to the emergency department, he was hypoxic, hypotensive, and in respiratory distress, prompting intubation and vasopressor support. EKG demonstrated ST-segment elevations in the anterolateral leads, raising concern for acute STEMI (Figure 1).

Laboratory studies revealed markedly elevated troponin, B-type natriuretic peptide (BNP), leukocyte, and procalcitonin levels, indicating acute myocardial injury and systemic inflammation (Table 1).

**Table 1:** Quantitative laboratory data obtained at presentation. Significant elevations in cardiac biomarkers, leukocyte count, and inflammatory markers are present. These values support the presence of acute myocardial injury and systemic inflammation.

Laboratory parameter	Value at presentation	Reference range
Troponin (ng/L)	1,700	<53
B-type natriuretic peptide (pg/mL)	345	<80
White blood cell count (cells/µL)	17,900	4,000–10,500
Procalcitonin (ng/mL)	7.06	<0.5

CT angiography (CTA) of the chest showed bilateral dependent infiltrates. Transthoracic echocardiography revealed severe left ventricular systolic dysfunction with an ejection fraction (LVEF) of 10-15% and a large wall motion abnormality in the proximal left anterior descending (LAD) artery distribution (Video 1).

**Video 1:** Initial echocardiography. Severe left ventricular systolic dysfunction is demonstrated with a left ventricular ejection fraction of 10-15%.

**Figure 2:**
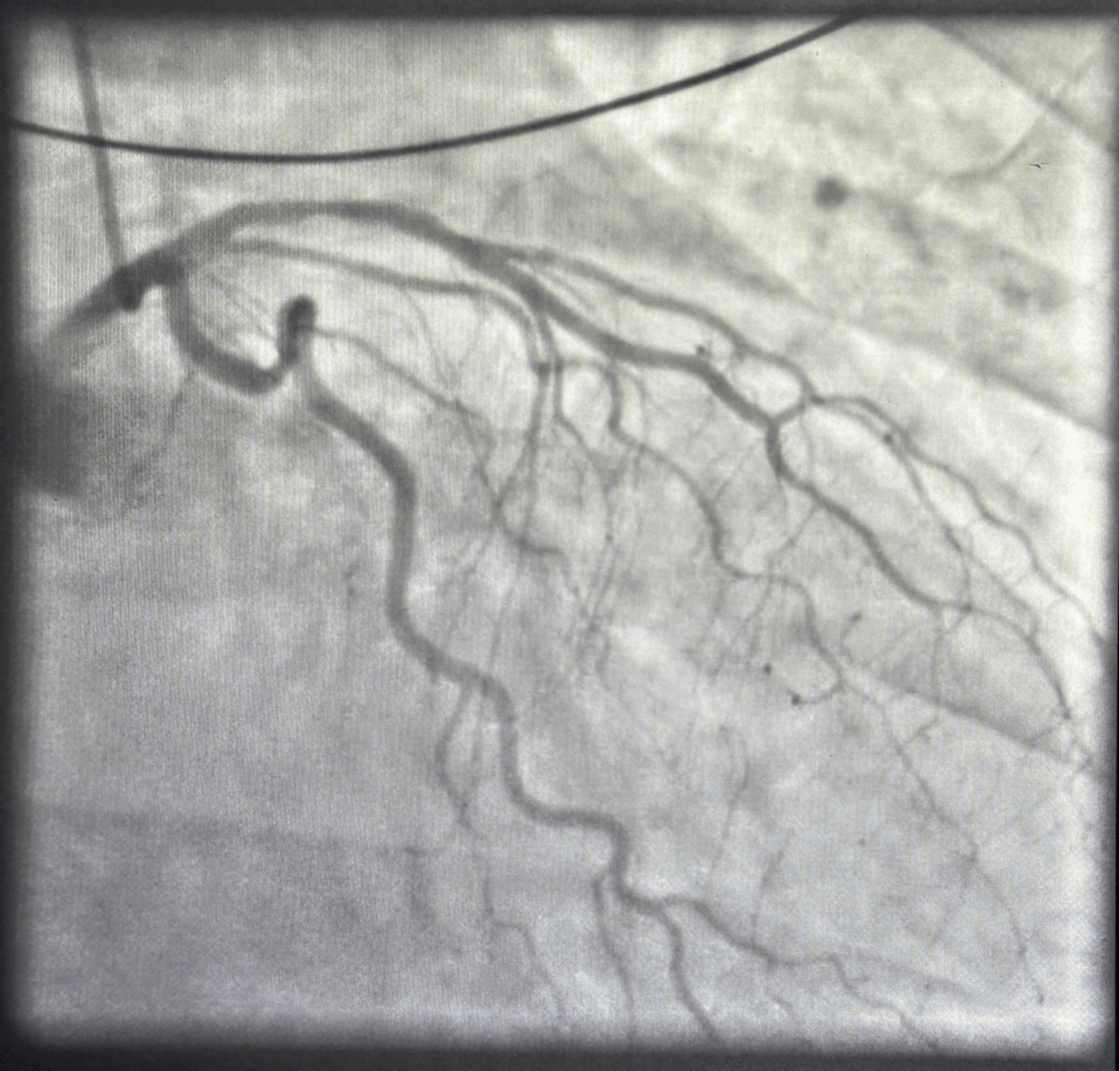
Coronary angiography. Coronary angiography demonstrates non-obstructive coronary artery disease, with moderate (approximately 40-50%) stenosis of the proximal left anterior descending artery, and no evidence of an acute culprit lesion, thrombus, or dissection. The remaining left coronary system shows preserved luminal caliber without significant obstructive disease.

Given the strong clinical suspicion for acute myocardial infarction, emergent coronary angiography was performed. The angiogram revealed non-obstructive coronary artery disease, including moderate (40-50%) stenosis of the proximal LAD artery, without evidence of an acute culprit lesion (Figure 2).

Right heart catheterization showed elevated right atrial and pulmonary capillary wedge pressures, with a left ventricular end-diastolic pressure of 30 mmHg. In the absence of significant coronary obstruction, alternative diagnoses such as viral myocarditis, sepsis-related cardiomyopathy, and Takotsubo syndrome were considered.

Given his infectious symptoms, without evidence of an obstructive coronary lesion, a comprehensive infectious workup was initiated. Empiric antibiotic therapy with omadacycline was started for presumed pneumonia based on pulmonary consolidation seen on CT imaging. Initial blood cultures grew coagulase-negative staphylococci, later interpreted as contaminants following persistently negative repeat cultures. Viral serologies returned positive for Coxsackie B virus, with elevated antibody titers to serotypes B1 through B6. The respiratory viral panel was also positive for parainfluenza virus. Given the fulminant clinical deterioration, the patient was diagnosed with mixed cardiogenic and septic shock. Circulatory support was continued with norepinephrine and placement of an intra-aortic balloon pump (IABP) to augment cardiac output.

Over the next 72 hours, the patient exhibited marked clinical improvement with stabilization of hemodynamics and resolution of respiratory failure, allowing for successful extubation. Vasopressor support was discontinued, guideline-directed medical therapy was initiated, and the IABP was removed. A repeat transthoracic echocardiogram on hospital day four demonstrated complete recovery of systolic function, with normalization of wall motion and an LVEF of 60% (Video 2).

**Video 2:** Repeat echocardiography on hospital day four. Recovery of systolic function is seen, with normalization of wall motion and a left ventricular ejection fraction.

The patient was discharged on carvedilol, lisinopril, and spironolactone and referred for outpatient cardiac rehabilitation. Given the rapid recovery, wearable defibrillator therapy and ICD placement were no longer indicated.

## Discussion

This case highlights the importance of considering viral myocarditis, including Coxsackie B infection, in patients presenting with ST-elevation and acute heart failure, and underscores the potential for a rapid full recovery with appropriate supportive treatment. It also highlights the diagnostic challenge posed by acute viral myocarditis, particularly when it presents with ST-segment elevations and signs of acute decompensated heart failure. The patient underwent emergent coronary angiography for suspected STEMI; however, the absence of obstructive coronary disease and subsequent positive serologic testing led to the diagnosis of Coxsackie B myocarditis.

Coxsackie B virus infection may present with hand-foot-and-mouth disease or with atypical systemic manifestations, including pleurodynia, pericarditis, and myocarditis [8]. Notably, Coxsackie B virus remains a common cause of viral myocarditis, inducing myocardial injury through both direct cytotoxic effects and immune-mediated mechanisms. It gains entry into cardiomyocytes via the coxsackie-adenovirus receptor (CAR), triggering an inflammatory cascade that results in tissue damage and impaired cardiac function [1]. Clinically, this often mimics myocardial infarction, with patients presenting with chest pain, ST-segment elevations, and elevated troponin levels. Although ST-segment elevation is generally diffuse in myocarditis, this can be territorial, mimicking a STEMI [4,5]. Several cases have also reported acute heart failure as a manifestation of Coxsackie B myocarditis [3,6]. In these situations, the clinician’s ability to synthesize clinical, imaging, and laboratory data is crucial to avoid unnecessary interventions and ensure appropriate management. The presence of fever and myalgias may serve as important clues pointing toward myocarditis as the underlying etiology [5].

In such presentations, distinguishing viral myocarditis from acute coronary syndrome is paramount. While cardiac MRI and endomyocardial biopsy are considered gold standards for diagnosing myocarditis, they are not always feasible [7]. In this case, as in others, serologic testing for viral pathogens proved pivotal in making the diagnosis [3,4].

Viral myocarditis is typically a self-limiting condition, with recovery occurring over weeks to months. However, the clinical course can be highly variable, ranging from complete recovery within days to persistent myocardial fibrosis and systolic dysfunction necessitating ICD placement [3,9,10]. Treatment for myocarditis is primarily supportive, focusing on guideline-directed management of heart failure and arrhythmias. No antiviral therapies are currently approved for viral myocarditis, though antivirals such as acyclovir or ganciclovir may be used empirically in suspected herpesvirus cases. In fulminant cases with cardiogenic shock, parenteral inotropes and temporary mechanical circulatory support (MCS) devices, such as IABP, veno-arterial extracorporeal membrane oxygenation, percutaneous ventricular assist devices, or Impella, may be needed as a bridge to recovery or transplantation. These devices vary in their hemodynamic effects, especially on afterload, which may influence inflammation. Patients experiencing ventricular arrhythmias during the acute phase are at higher risk for recurrence and may require ICD placement [3]. In some reports, levosimendan has been used to support cardiac output in severe cases [11].

Ultimately, this case underscores the importance of maintaining a broad differential diagnosis in patients presenting with myocardial injury and non-obstructive coronary arteries. It also highlights the value of early repeat echocardiography in cases of suspected myocarditis, particularly to assess for recovery of cardiac function when considering the need for defibrillator therapy before discharge.

## Conclusions

This case illustrates the potential for Coxsackie B viral myocarditis to closely mimic acute STEMI, presenting with cardiogenic shock and severe systolic dysfunction. Prompt recognition, supported by a high index of suspicion and confirmatory serologic testing, was essential in establishing the diagnosis. Supportive management, including pharmacologic therapy and mechanical circulatory support such as an IABP, can lead to significant improvement and, in some cases, rapid normalization of ventricular function. This case underscores the importance of considering viral myocarditis in the differential diagnosis of acute cardiac presentations, particularly when coronary angiography fails to reveal obstructive disease and infectious symptoms such as fever or myalgia are present. It also highlights the value of serologic testing when cardiac MRI or biopsy is not feasible. 
